# Comparison of Ga-68 PSMA positron emission tomography/computerized tomography with Tc-99m MDP bone scan in prostate cancer patients

**DOI:** 10.3906/sag-1807-4

**Published:** 2019-02-11

**Authors:** Lebriz USLU-BEŞLİ, Sait SAĞER, Elife AKGÜN, Sertaç ASA, Onur Erdem ŞAHİN, Çetin DEMİRDAĞ, Ekrem GÜNER, Baresh Razavi KHOSROSHAHI, Emre KARAYEL, Hüseyin PEHLİVANOĞLU, Aslan AYGÜN, İlhami USLU, Zübeyr TALAT, Kerim SÖNMEZOĞLU

**Affiliations:** 1 Department of Nuclear Medicine, Cerrahpaşa Medical Faculty, İstanbul University-Cerrahpaşa, İstanbul Turkey; 2 Department of Urology, Cerrahpaşa Medical Faculty, İstanbul University-Cerrahpaşa, İstanbul Turkey; 3 Department of Urology, Health Sciences University Bakırköy Sadi Konuk Training and Research Hospital, İstanbul Turkey; 4 Division of Radiopharmacy, Department of Nuclear Medicine,Cerrahpaşa Medical Faculty,İstanbul University-Cerrahpaşa, İstanbul Turkey

**Keywords:** Ga-68 PSMA, PET/CT, bone scintigraphy, prostate cancer, bone metastasis

## Abstract

**Background/aim:**

The aim of our study was to compare Tc-99m MDP bone scan and Ga-68 PSMA PET/CT in terms of detection of bone metastasis in prostate cancer patients.

**Materials and methods:**

A total of 28 prostate cancer patients with bone scan and PSMA PET/CT performed within 90 days were retrospectively included in our analysis. All bone lesions were scored as negative (score-0), positive (score-1), or suspicious (score-2) for metastasis by two experienced nuclear medicine physicians. Both patient-based and region-based analyses were made for all osseous lesions.

**Results:**

On per-patient analysis; sensitivity, specificity, positive predictive value (PPV), negative predictive value (NPV), and accuracy were 72.7%, 52.9%, 50%, 75%, and 60.7%, respectively, for bone scan and 90.9%, 100%, 100%, 94.4%, and 96.4%, respectively, for PSMA PET/CT. On per-region analysis; sensitivity, specificity, PPV, NPV, and accuracy were 76.2%, 80.9%, 57.1%, 91.1%, and 79.8%, respectively, for bone scan and 85.7%, 100%, 100%, 95.5%, and 95.4%, respectively, for PSMA PET/CT.

**Conclusion:**

Ga-68 PSMA PET/CT has higher sensitivity, specificity, and accuracy compared to bone scan in terms of bone metastasis detection in prostate cancer patients. Therefore, it might be the modality of choice for patients with suspicion for metastatic disease, despite negative bone scan and conventional imaging results.

## 1. Introduction

Prostate cancer is the second most common cancer in men worldwide after lung cancer and the sixth leading cause of cancer-related death [1]. Prostate cancer generally originates from peripheral zone of the prostate gland. The most common prognostic indicator of prostate cancer is Gleason score and stage of the disease. Prostate-specific antigen (PSA) is a glycoprotein produced by prostate cells, including prostate cancer cells, and serum PSA level measurement provides disease monitoring, staging, and early detection of prostate cancer [2]. Transrectal ultrasonography (TRUS) is generally performed initially in patients with elevated PSA levels and suspicious transrectal digital examination (DRE) result. The diagnosis of prostate cancer is generally made by prostate core needle biopsy under TRUS guidance [3]. Multiparametric prostate MRI (mp-MRI) is a novel imaging approach for diagnosis and localization of primary prostate lesions and can guide targeted prostate biopsy [4]. Computerized tomography (CT) has limited value in staging of prostate cancer due to very low sensitivity [5]. Tc-99m methylene diphosphonate (Tc-99m MDP) bone scan is a sensitive method for detection of prostate bone metastasis; however, it has limited specificity and many benign bone lesions can cause false-positive bone scan results [6].

Prostate-specific membrane antigen is a glutamate carboxypeptidase-II, which is overexpressed in prostate cancer cells [7]. Ga-68-labeled urea-based PSMA inhibitor (Ga-68-PSMA-HBED-CC) is a novel positron emission tomography (PET) tracer for staging of prostate cancer patients with a high accuracy for detection of lymph node and organ metastasis, as well as for detection of residual or recurrent local disease [5]. PSMA ligands can also be labeled with Lu-177, which is a gamma and beta-emitter radionuclide, enabling radionuclide therapy for Ga-68 PSMA positive tumor foci [8]. 

Skeletal system is the most common site for distant organ metastasis in prostate cancer patients and 5-year survival decreases to about 30% when distant metastases have occurred [9]. Bone scan is a sensitive and relatively inexpensive method for detection of bone metastasis in prostate cancer patients; however, it has limited specificity and many benign bone diseases can mimic metastasis on bone scan.

Ga-68 PSMA PET is relatively a new diagnostic modality, but it has gained widespread importance due to its excellent diagnostic performance in detection of lymph node and organ metastasis, as well as in detection of primary tumor [10,11] and leads to change in management in at least 50% of patients with biochemical recurrence [12]. Ga-68 PSMA PET/CT was shown to detect metastasis even in patients with low PSA values [13]. However, to date, there are limited number of studies comparing Ga-68 PSMA PET/CT with bone scan in prostate cancer patients. 

The aim of our study was to compare Tc-99m MDP bone scan and Ga-68 PSMA positron emission tomography/computerized tomography (PET/CT) in terms of bone metastasis detection in prostate cancer patients. 

## 2. Materials and methods

### 2.1. Patient selection

Biopsy- or postoperative histopathology-proven prostate cancer patients with Ga-68 PSMA PET/CT and Tc-99m MDP bone scan performed within 90 days either for initial staging or for restaging of the disease between March 2015 and March 2016 were retrospectively included in our analysis. Gleason scores were ≥7, except for three patients with biopsy-proven Gleason score of 3 + 3, clinical suspicion for metastatic disease, and increasing serum PSA levels. Patients did not receive any treatment between the two scans other than androgen deprivation therapy. Gleason scores as well as serum PSA levels at the time of imaging were available for all patients. 

### 2.2. Imaging protocol

For PET/CT imaging, all patients were injected intravenously with a mean activity of 92.5–148 MBq (2.5–4 mCi) Ga-68-HBED-CC. Radiolabeling procedure was performed using a fully automated radiopharmaceutical synthesis device based on a modular concept (Eckert & Ziegler Eurotope, Berlin, Germany) as described previously by Kabasakal et al. [14]. All Ga-68 PSMA PET/CT images were acquired using an integrated PET/CT scanner (Siemens Biograph 6, Knoxville, TN, USA or GE Discovery 710, Waukesha, WI, USA) at 45 min postinjection. An initial CT topogram was followed by a CT transmission scan and an emission PET scan from vertex to midthigh. Imaging parameters for transmission CT scan were as follows: Low tube current (130 kVp 48–76 mAs), slice thickness of 4.0 mm, gantry rotation time of 0.6 s and collimator width of 6 × 3 mm. PET emission scan was acquired at 3 min per bed position at caudocranial direction. Iterative image reconstruction method using CT transmission images were utilized for attenuation correction. All patients were asked to empty their bladder before initiation of PET/CT acquisition to minimize bladder activity. 

Bone scan was performed by intravenous injection of 740-1110 MBq (20–30 mCi) Tc-99m MDP. Planar and SPECT or SPECT/CT bone imaging were available. Planar and SPECT/CT imaging was acquired using an integrated SPECT/CT scanner (Siemens Symbia T16, Hoffman Estates, IL, USA) in six patients. SPECT/CT was performed around foci of suspicious uptake at whole-body scan using imaging parameters of a low-dose CT scan with an effective tube current time of 30–40 mAs, pitch 0.65, gantry rotation time of 1.0 s, tube voltage of 110 kV, slice thickness of 5 mm. For 22 patients, who had prior bone scan in another hospital within 90 days, available scintigraphic images were included for analysis.

### 2.3. Image analysis

All PET/CT images were reviewed and analyzed by two nuclear medicine physicians separately using vendor-based work station (GE AW Volume Share 5, GE Medical Systems). All lesions with initial disagreement between readers were reevaluated together with both readers to conclude in a final common consensus. Both patient-based and region-based analyses (vertebrae, extremities, and all the other bones analyzed separately) were made for osseous lesions detected by Ga-68 PSMA PET/CT and bone scan. The data were scored as negative for metastasis (score-0), positive for metastasis (score-1), and suspicious for metastasis (score-2). The gold standard was accepted as follow-up imaging, including Ga-68 PSMA PET, bone scintigraphy, CT or MRI, as well as clinical follow-up for at least 1 year. 

### 2.4. Statistical analysis

All data was compared using the McNemar test. Sensitivities, specificities, positive and negative predictive values and accuracies were calculated for both Ga-68 PSMA PET/CT and bone scan, counting both score-1 and score-2 as positive for metastasis.

## 3. Results

A total of 28 prostate cancer patients with a mean age of 67.3 ± 7.4 years and a mean serum PSA value of 25.49 ± 32.7 ng/mL (median: 9.39 ng/mL, range: 0.5–125.1 ng/mL) were included in our study. Seven patients had a Gleason score of 3 + 4, 7 had 4 + 3, 6 had 4 + 4, 3 had 4 + 5, and 2 had 5 + 4. Three patients had biopsy-proven Gleason score of 3 + 3, but had elevated serum PSA levels (3.1, 14.3, 75.1 ng/mL) and clinical suspicion for metastatic disease. Thirteen patients with a Gleason score of ≥7 had a prior prostatectomy operation. Patient characteristics are given in Table 1.

**Table 1:** Patient characteristics.

Patient no.	Age (years)	PSA level (ng/mL)	Gleason score	Local recurrence	Lymph node metastasis
1	69	0.45	3 + 4	-	-
2	61	3.1	3 + 3	-	-
3	70	6.85	4 + 4	+	-
4	66	17.13	4 + 3	+	+
5	71	38.93	5 + 4	+	-
6	58	75.10	3 + 3	-	-
7	65	8.50	4 + 4	-	-
8	68	8.10	4 + 5	-	-
9	60	5.2	4 + 3	-	-
10	72	14.3	3 + 3	+	-
11	70	0.07	4 + 4	-	-
12	54	65.44	5 + 4	+	+
13	71	1.60	4 + 4	-	-
14	82	39.38	3 + 4	+	-
15	74	93.65	4 + 3	+	+
16	76	10.28	4 + 3	+	-
17	61	23.53	4 + 4	+	-
18	66	4.19	3 + 4	+	-
19	73	4.98	4 + 4	+	+
20	78	57.18	4 + 5	+	-
21	66	66.33	3 + 4	+	-
22	70	0.39	3 + 4	-	-
23	49	0.24	3 + 4	-	-
24	73	8.36	4 + 3	+	-
25	68	6.29	3 + 4	+	-
26	62	125.06	4 + 3	+	-
27	73	12.09	4 + 3	+	+
28	58	20.16	4 + 5	-	+

On Ga-68 PSMA PET/CT, 16 patients (57.1%) had local recurrent tumor focus on prostatic bed, whereas 12 patients (42.9%) did not have any local PSMA uptake. Seven patients (25%) had additional lymph node metastasis on Ga-68 PSMA PET/CT. 

**Figure 1:**
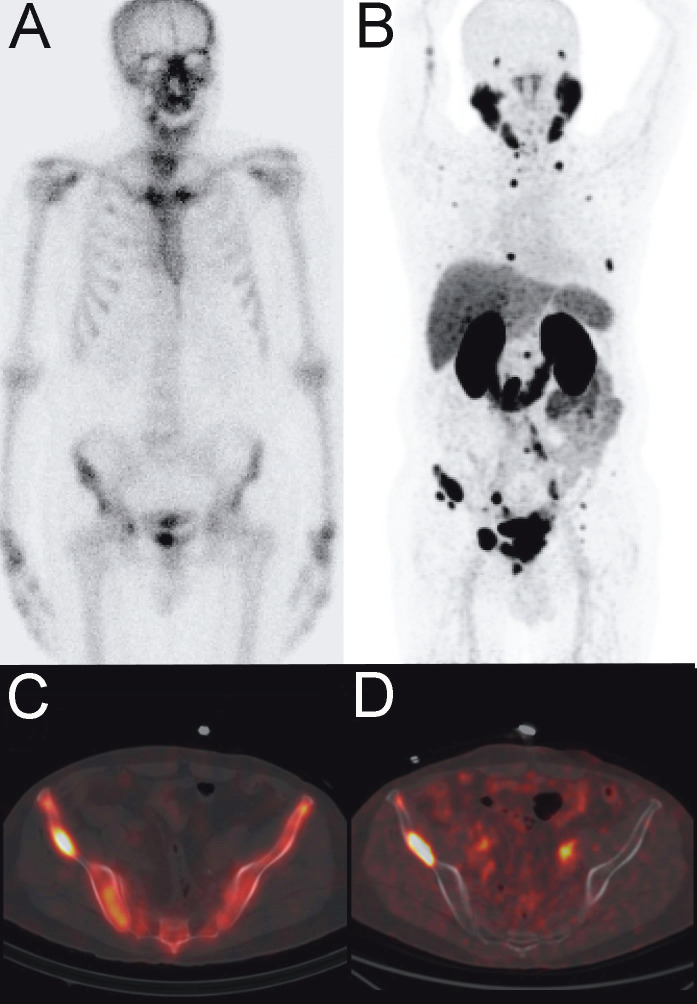
Tc-99m MDP bone scintigraphy planar (A), and fused SPECT/CT (C) images compared to Ga-68 PSMA PET maximum intensity projection (MIP) (B) and fused PET/CT (D) images in a prostate cancer patient with a Gleason score of 4 + 3 and serum PSA level of 17.13 ng/mL. In addition to several bone lesions that are concomitantly positive on bone scan and PSMA PET images (C and D), PSMA PET MIP image shows many other bone metastasis that are false-negative on bone scan (A and B).

**Figure 2:**
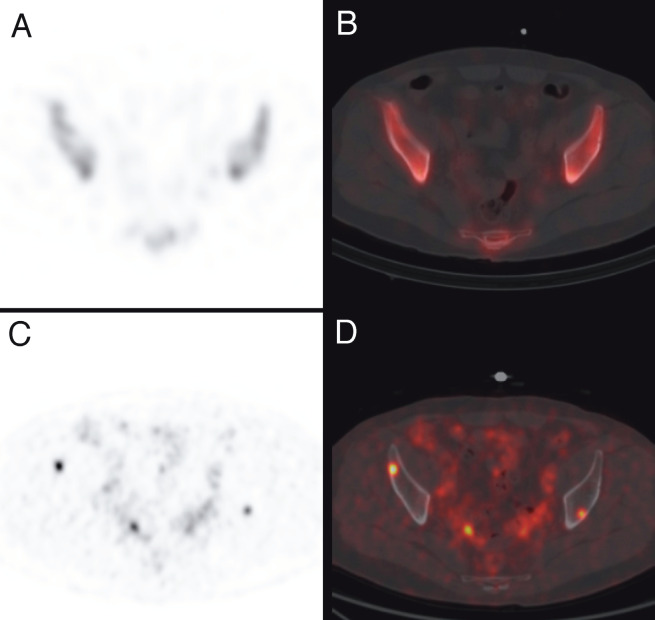
Tc-99m MDP bone scintigraphy SPECT (A), fused SPECT/CT (B), Ga-68 PSMA axial PET (C), and fused PET/CT (D) images of the same prostate cancer patient. Ga-68 PSMA PET shows additional bone metastasis that are false negative on bone SPECT and SPECT/CT.

On per-patient analysis, 9 patients were score-0 and 5 patients were score-1 concomitant on both Ga-68 PSMA PET/CT and bone scan and the gold standard confirmed true-negative and true-positive results, respectively (Table 2) (Figures 1 and 2). Among the remaining 14 patients, Ga-68 PSMA PET/CT could successfully detect bone metastasis that was missed by bone scan in 3 patients (patients 3, 5, 23) and correctly excluded presence of metastasis that was recorded as score-1 (patients 1, 18, 25) or score-2 (patient 10) on bone scan in 4 patients. Two patients were recorded as score-2 on bone scan and score-1 on Ga-68 PSMA PET/CT and the gold standard confirmed presence of metastasis on both patients (patients 14 and 28). When both score-1 and score-2 are counted as positive for metastasis, sensitivity, specificity, positive predictive value, negative predictive value, and accuracy of bone scan are 72.7%, 52.9%, 50%, 75%, and 60.7%, whereas for Ga-68 PSMA PET/CT they are found as 90.9%, 100%, 100%, 94.4%, and 96.4%, respectively (Table 3).

**Table 2:** Patient-based comparison of Ga-68 PSMA PET/CT, Tc- 99m MDP bone scan, and gold standard. 0 = score-0 (negative for metastasis), 1 = score-1 (positive for metastasis), 2 = score-2 (suspicious for metastasis).

Patient no.	Bone scan	Ga-68 PSMA PET/CT	Gold standard
1	2	0	0
2	0	0	0
3	0	1	1
4	1	1	1
5	0	1	1
6	1	0	0
7	0	0	0
8	1	1	1
9	2	0	0
10	2	0	0
11	1	1	1
12	2	0	0
13	0	0	0
14	2	1	1
15	1	1	1
16	2	0	0
17	0	0	0
18	1	0	0
19	0	0	0
20	0	0	0
21	0	0	0
22	0	0	0
23	0	1	1
24	0	0	0
25	1	0	0
26	1	1	1
27	1	0	1
28	2	1	1

**Table 3:** Sensitivity, specificity, accuracy, positive predictive value, and negative predictive value of Ga-68 PSMA PET and Tc-99m MDP bone scan on per-patient analysis. TP: true-positive, TN: true-negative, FP: false-positive, FN: false-negative, PPV: positive predictive value, NPV: negative predictive value.

	TP	TN	FP	FN	Sensitivity	Specificity	PPV	NPV	Accuracy
Bone scan	8 (28.6%)	9 (32.1%)	8 (28.6%)	3 (10.7%)	72.7%	52.9%	50%	75%	60.7%
Ga-68 PSMA PET/CT	10 (35.7%)	17 (60.7%)	0 (0%)	1(3.6%)	90.9%	100%	100%	94.4%	96.4%

**Figure 3:**
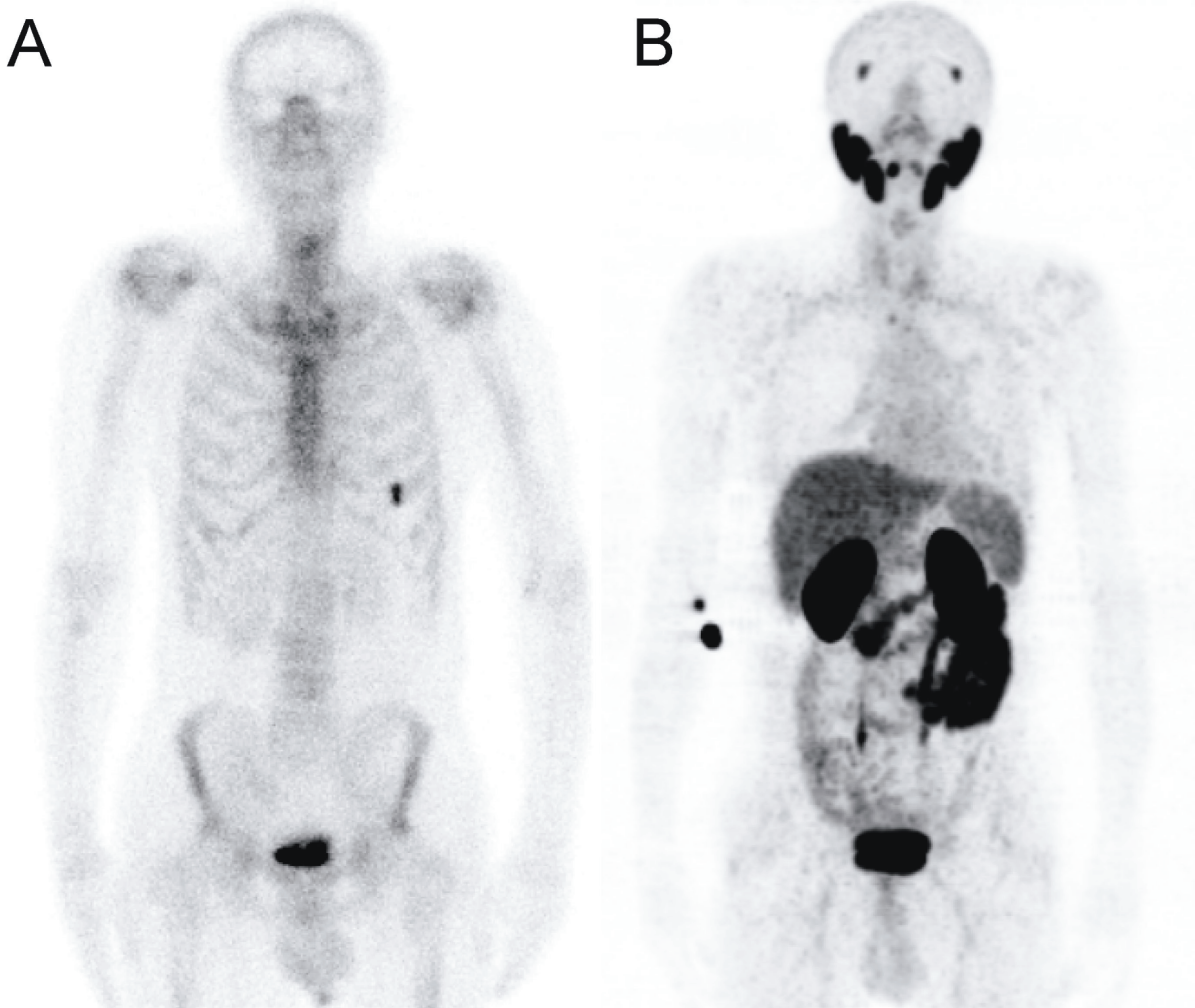
Tc-99m MDP bone scintigraphy planar image (A) compared to Ga-68 PSMA PET maximum intensity projection (MIP) images (B) in a prostate cancer patient with a Gleason score of 4 + 4 and serum PSA level of 0.7 ng/mL. Bone scan shows a focal uptake on anterior part of left sixth rib, which was negative on PSMA PET.

**Figure 4:**
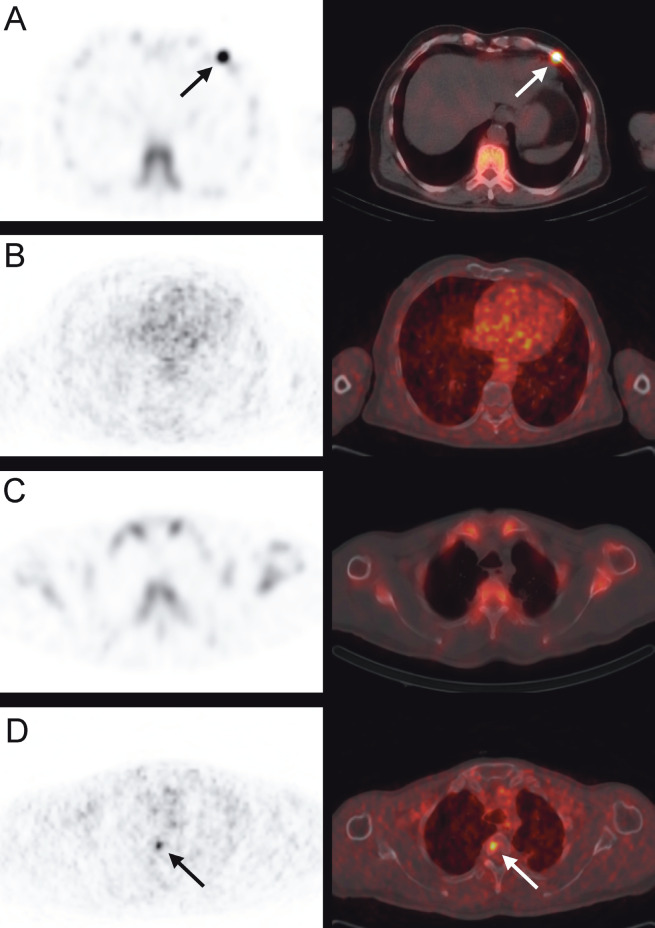
Tc-99m MDP bone scintigraphy SPECT/CT (A and C) and Ga-68 PSMA PET/CT (B and D) images of the same prostate cancer patient. While the rib lesion with increased uptake on bone scan (A) is negative on PSMA PET/CT (B), PSMA PET/CT shows additional uptake on third dorsal vertebra (D) (arrow), which is negative on bone scan (C).

On per-region analysis, 19 patients had at least one positive lesion in at least one region in either of the imaging modalities (Table 4). When only vertebral region is analyzed, 7/19 patients were concomitantly score-0 and 4/19 patients were concomitantly score-1. Among the remaining 8 patients, 6 were score-1 or score-2 on bone scan, while Ga-68 PSMA PET/CT was true-negative. One patient was false-negative on bone scan and true-positive on Ga-68 PSMA PET/CT (patient 11) (Figures 3 and 4). On the contrary, one patient was true-positive on bone scan and false-negative on Ga-68 PSMA PET/CT.

**Table 4:** Region-based comparison of Ga-68 PSMA PET/CT, Tc-99m MDP bone scan, and gold standard. 0 = score-0 (negative for metastasis), 1 = score-1 (positive for metastasis), 2 = score-2 (suspicious for metastasis).

Patient no.	Vertebral column Bone scan	Vertebral column PSMA PET	Vertebral column Gold standard	Extremities Bone scan	Extremities PSMA PET	Extremities Gold standard	Others Bone scan	Others PSMA PET	Others Gold standard
1	2	0	0	0	0	0	0	0	0
3	0	0	0	0	0	0	0	1	1
4	1	1	1	0	0	0	1	1	1
5	0	0	0	0	0	0	0	1	1
6	1	0	0	0	0	0	1	0	0
8	1	1	1	1	1	1	1	1	1
9	0	0	0	0	0	0	2	0	0
10	2	0	0	0	0	0	0	0	0
11	0	1	1	0	0	0	1	0	1
12	0	0	0	0	0	0	2	0	0
14	0	0	0	2	1	1	0	1	1
15	1	1	1	1	1	1	1	1	1
16	2	0	0	2	0	0	0	0	0
18	1	0	0	0	0	0	1	0	0
23	0	0	0	0	0	0	0	1	1
25	1	0	0	0	0	0	1	0	0
26	1	1	1	1	1	1	1	1	1
27	1	0	1	0	0	0	1	0	1
28	0	0	0	0	0	0	2	1	1

When extremities are analyzed, 14 patients were concomitantly score-0 and 3 patients were concomitantly score-1. One was score-2 on bone scan and score-0 on Ga-68 PSMA PET/CT and the gold standard confirmed absence of metastasis (patient 16). In another patient (patient 14), bone scan was score-2 while Ga-68 PSMA PET/CT was score-1 and the gold standard confirmed presence of metastasis.

On per-region analysis, when both score-1 and score-2 are counted as positive for metastasis, sensitivity, specificity, positive predictive value, negative predictive value, and accuracy of bone scan are 76.2%, 80.9%, 57.1%, 91.1%, and 79.8%, whereas for Ga-68 PSMA PET/CT they are found as 85.7%, 100%, 100%, 95.5%, and 95.4%, respectively (Table 5).

**Table 5:** Sensitivity, specificity, accuracy, positive predictive value, and negative predictive value of Ga-68 PSMA PET and Tc-99m MDP bone scan on per-region analysis. TP: true-positive, TN: true-negative, FP: false-positive, FN: false-negative, PPV: positive predictive value, NPV: negative predictive value.

	TP	TN	FP	FN	Sensitivity	Specificity	PPV	NPV	Accuracy
Bone scan	16 (19.0%)	51 (60.7%)	12 (14.3%)	5 (5.6%)	76.2%	80.9%	57.1%	91.1%	79.8%
Ga-68 PSMA PET/CT	18 (21.4%)	63 (75%)	0 (0%)	3 (3.6%)	85.7%	100%	100%	95.5%	96.4%

## 4.Discussion

Pyka et al. had the largest cohort comprising a total of 126 prostate cancer patients for comparison of Ga-68 PSMA PET and bone scan and to our knowledge, it is the only study present comparing sensitivity and specificity of both imaging in the same cohort [15]. On patient-based analysis, sensitivity and specificity of Ga-68 PET were found as 98.7%–100% and 88.2%–100%, respectively, depending on the positive or negative classification of equivocal lesions, whereas the sensitivity and specificity of bone scan were 86.7%–89.3% and 60.8%–96.1%, respectively. On region-based analysis, sensitivity and specificity were found as 98.8%–99.0% and 98.9%–100% for Ga-68 PSMA PET/CT and 82.4%–86.6% and 91.6%–97.9% for bone scan, respectively. Janssen et al. compared Ga-68 PSMA PET/CT with Tc-99m diphosphono-1,2-propanodicarboxylic acid (Tc-99m DPD) bone scan including SPECT/CT in 54 prostate cancer patients and found both sensitivity and specificity of Ga-68 PSMA PET/CT as 100%, while they were 82.8% and 84% for Tc-99m DPD SPECT/CT [16]. There are also other studies comparing Ga-68 PSMA PET and bone scan, all with limited number of patients. Kabasakal et al. revealed that bone scan and Ga-68 PSMA PET/CT were concurrently positive for metastasis in four out of 25 patients with bone scan [14]. However, nine out of 25 prostate cancer patients were found to be suspicious for metastasis on bone scan, although Ga-68 PSMA PET/CT confirmed metastasis only in one of them and revealed additional bone metastasis in two patients with normal bone scan. 

On per-patient analysis, our results revealed sensitivity and specificity of 90.9% and 100% for Ga-68 PSMA PET/CT and 72.7% and 52.9% for bone scan. On per-region analysis, our sensitivity and specificity were 85.7% and 100% for Ga-68 PET/CT and 76.2% and 80.9% for bone scan, respectively. Our sensitivity and specificity for Ga-68 PSMA PET/CT is comparable with the current literature; however, they are lower in the case of bone scan, probably due to lack of SPECT/CT in all of our patients. Also, our cohort of patients were referred to our department for Ga-68 PET/CT due to biochemical recurrence or suspicious bone lesions which could be the reason for lower sensitivity and specificity of bone scan. 

Both presence and extent of bone metastasis are important prognostic factors. Therefore, proper detection of presence and localization of bone metastasis is important in treatment strategy of prostate cancer patients [17,18]. In our cohort of 28 patients, Ga-68 PSMA PET/CT changed management of treatment in 7 patients by confirming presence of bone metastasis that was missed by bone scan in 3 patients or by excluding presence of metastasis that was recorded on bone scan in 4 patients. Also, Ga-68 PSMA PET/CT could guide Lu-177 PSMA therapy in patients with multiple metastases exhibiting PSMA uptake.

The main limitations of our study would be retrospective data, limited number of patients, lack of available SPECT/CT images for all patients, and lack of comparison of planar images with SPECT or SPECT/CT images for bone scan. Due to the limited number of patients, we could not perform separate statistical analysis involving PSA values or Gleason scores. Also, further studies would be necessary to compare the impact of Ga-68 PSMA PET/CT and bone scan in terms of change in treatment plan and prognosis of patients.

In conclusion, our results reveal that Ga-68 PSMA PET/CT has higher sensitivity, specificity and accuracy compared to bone scan in terms of bone metastasis in prostate cancer patients. Knowing that Ga-68 PSMA PET/CT would not be cost-effective to be performed in all prostate cancer patients, it might be the modality of choice for patients with clinical or biochemical suspicion for metastatic disease, despite negative bone scan and conventional imaging results.
